# Broccoli sprout extract prevents diabetic cardiomyopathy via Nrf2 activation in *db*/*db* T2DM mice

**DOI:** 10.1038/srep30252

**Published:** 2016-07-26

**Authors:** Zheng Xu, Shudong Wang, Honglei Ji, Zhiguo Zhang, Jing Chen, Yi Tan, Kupper Wintergerst, Yang Zheng, Jian Sun, Lu Cai

**Affiliations:** 1 Cardiovascular Center, the First Hospital of Jilin University, Changchun, China; 2 Kosair Children’s Hospital Research Institute, Department of Pediatrics, University of Louisville, Louisville, KY, USA; 3 Wendy Novak Diabetes Care Center, University of Louisville, Louisville, KY, USA; 4 Division of Endocrinology, Department of Pediatrics, the University of Louisville, Louisville, KY, USA

## Abstract

To develop a clinic-relevant protocol for systemic up-regulation of NFE2-related factor 2 (Nrf2) to prevent diabetic cardiomyopathy (DCM), male *db*/*db* and age-matched wild-type (WT) mice were given sulforaphane (SFN, an Nrf2 activator) and its natural source, broccoli sprout extract (BSE) by gavage every other day for 3 months, with four groups: vehicle (0.1 ml/10 g), BSE-low dose (estimated SFN availability at 0.5 mg/kg), BSE-high dose (estimated SFN availability at 1.0 mg/kg), and SFN (0.5 mg/kg). Cardiac function and pathological changes (hypertrophy, fibrosis, inflammation and oxidative damage) were assessed by echocardiography and histopathological examination along with Western blot and real-time PCR, respectively. Both BSE and SFN significantly prevented diabetes-induced cardiac dysfunction, hypertrophy and fibrosis. Mechanistically, BSE, like SFN, significantly up-regulated Nrf2 transcriptional activity, evidenced by the increased Nrf2 nuclear accumulation and its downstream gene expression. This resulted in a significant prevention of cardiac oxidative damage and inflammation. For all these preventive effects, BSE at high dose provided a similar effect as did SFN. These results indicated that BSE at high dose prevents DCM in a manner congruent with SFN treatment. Therefore, it suggests that BSE could potentially be used as a natural and safe treatment against DCM via Nrf2 activation.

Diabetic complications are among the most challenging health problems today. Cardiovascular complications, including diabetic cardiomyopathy (DCM), account for more than 80% of diabetic deaths^1^. Researchers seek new therapeutic targets to slow or reverse diabetes because of the growing incidence and the increased risk of mortality due to its complications. Several mechanisms responsible for DCM have been proposed^2^,^3^,^4^: (a) impaired regulation of intracellular calcium, leading to impaired cardiac contractility; (b) mitochondrial dysfunction, leading to over-production of reactive oxygen or nitrogen species (ROS, RNS), and even cardiac cell death; (c) abnormal cellular metabolism, leading to accumulation of toxic lipids in the heart; (d) accumulation of advanced glycated end-products in the heart, leading to extracellular matrix accumulation that in turn causes cardiac diastolic dysfunction and eventually functional failure. All these pathogenic factors could contribute to DCM to a certain extent, but all of these effects are thought to be related to oxidative stress^2^,^4^,^5^,^6^,^7^.

Oxidative stress arises out of an imbalance between ROS and/or RNS generation, and their clearance by antioxidant defense systems^4^. Due to low basal levels of antioxidants in the normal heart compared to other organs, the heart is highly susceptible to oxidative stress and damage^8^. Diabetes not only generates extra ROS/RNS but also impairs antioxidant capacity in the heart^4^,^5^. Therefore, enhancing cardiac endogenous antioxidant capacity is an attractive target for drug development to prevent DCM^4^,^9^.

Transcriptional regulation of the genes encoding antioxidants is controlled, in part, through antioxidant response elements (ARE)^10^,^11^. As one of the cap ‘n’ collar family members, Nrf2 is an important regulator of cellular detoxification responses and redox status^12^ and can lead to ARE-mediated basal and inducible expression of more than 200 genes. These genes can be grouped into several categories, including antioxidant genes and phase II detoxifying enzymes, such as NADPH quinone oxidoreductase (NQO1), hemeoxygenase-1 (HO-1), glutathione S-transferase, superoxide dismutase (SOD), catalase (CAT), and γ-glutamylcysteine synthetase^11^,^13^. Therefore, Nrf2 activators have become an attractive approach for the prevention of diabetic complications^14^,^15^.

Sulforaphane (SFN) is a molecule within the isothiocyanate group of organosulfur compounds from cruciferous vegetables, such as broccoli, brussel sprouts or cabbage^16^,^17^. As a potent Nrf2 activator, SFN has been reported to prevent various diabetic complications in several animal models^18^,^19^,^20^,^21^,^22^. To our knowledge, however, the effects of SFN directly used in human subjects on the prevention of diabetic complications have never been reported.

Recently, several pre-clinical and clinical trials have explored the preventive effect of broccoli sprout preparation on diseases such as chronic obstructive pulmonary disease^10^ and asthma^23^,^24^, and have also assessed the tolerance and effect in healthy volunteers^25^,^26^,^27^,^28^. Unfortunately, there are no available data on the preventive effects of broccoli sprout preparation on diabetic cardiovascular complications. Therefore, this study aimed to evaluate whether the chronic use of broccoli sprout extract (BSE), a natural SFN-rich supplement, can prevent the development of DCM.

In this study, transgenic *db*/*db* mice were used as an animal model of type 2 diabetes mellitus (T2DM). Diabetic and age matched wild-type (WT) mice were treated with BSE and SFN for 3 months. We found that BSE up-regulated Nrf2 expression and transcription in the heart and significantly prevented the development of DCM.

## Results

### General features of mouse model

**Figure 1:**
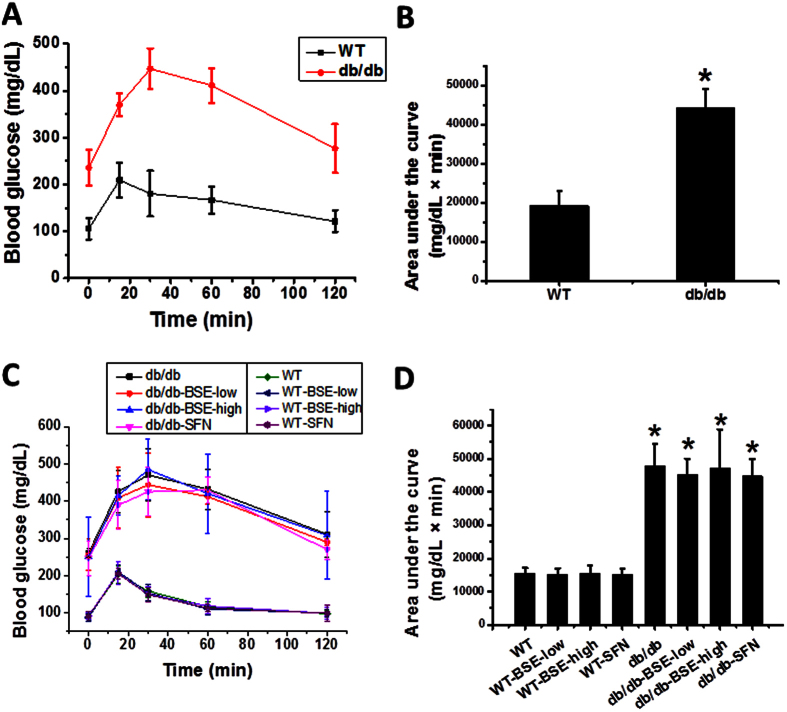
Effect of BSE and SFN on glucose tolerance in *db*/*db* and WT mice models. IPGTT (glucose 2 g/kg, bodyweight) were performed before (**A**,**B**) and after 3 months of treatment (**C**,**D**), follow by calculating the integrated area under the curves (AUC) (**A**–**D**).

Both *db*/*db* and WT mice were fed with BSE or SFN every other day for 3 months. BSE or SFN treatment did not significantly changed the body weight among the groups (Data not shown). Before and after BSE treatment, glucose tolerance was assessed using a standard intraperitoneal glucose tolerance test (IPGTT) followed by calculation of the area under the curve (AUC). Before treatments, *db*/*db* mice showed a significant increase in glucose tolerance (Fig. 1A,B). Three-month treatments of WT and *db*/*db* mice with either BSE or SFN did not affect fasting blood glucose levels (the data at time 0), nor did it affect the glucose tolerance among *db*/*db* and WT groups, separately (Fig. 1C,D).

### BSE and SFN prevented diabetes-induced cardiac dysfunction in T2DM mice

To define whether BSE and SFN protect against diabetes-induced cardiac dysfunction in *db*/*db* mice, we examined cardiac function by Echo and found that *db*/*db* mice had significantly decreased left ventricular (LV) internal systolic diameter (LVID; s), LV end systolic volume (LV vol; s), LV mass, ejection fraction (EF) and fractional shortening (FS) (Table 1). Treatment of these mice with BSE and SFN largely and significantly prevented the development of these cardiac dysfunctions. The effect of BSE-high dose was stronger than BSE-low dose, and approached the effect of SFN.

### BSE and SFN prevented diabetes-induced histopathological changes in *db*/*db* mice

**Figure 2:**
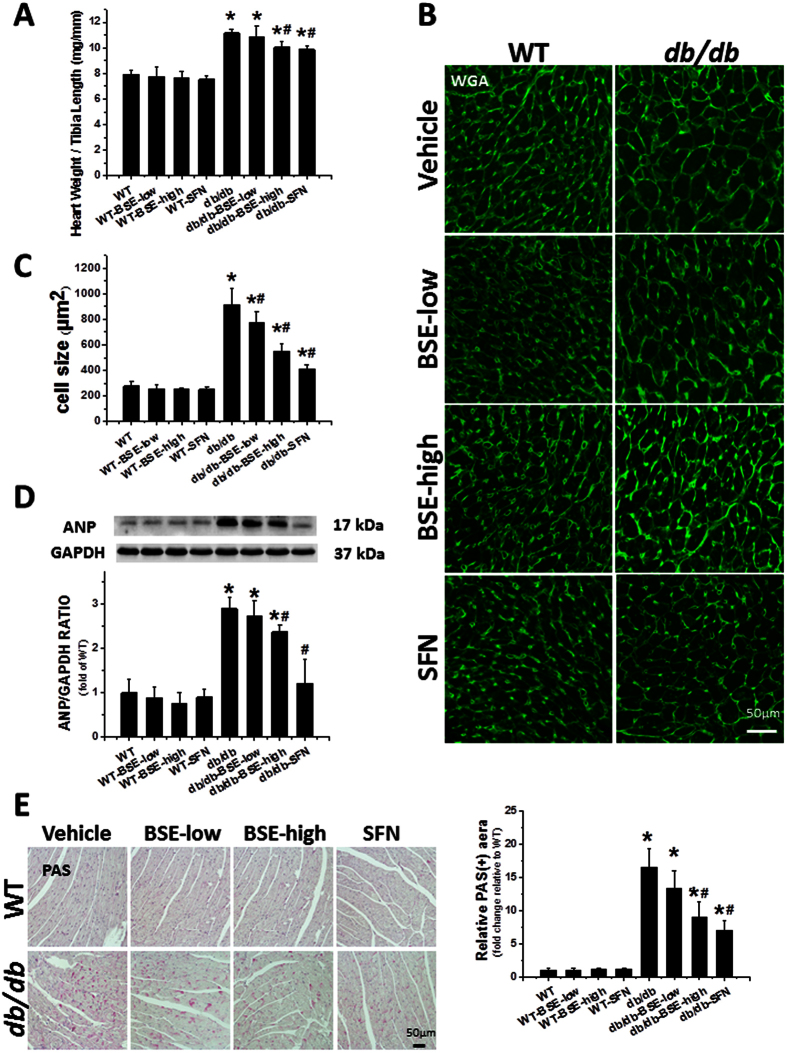
BSE and SFN prevented diabetes-induced cardiac hypertrophy and glycogen accumulation. *db*/*db* and age-matched WT mice were fed with BSE or SFN every other day for 3 months. After 3 months of treatment, mice were euthanatized, then the ratio of heart weight to tibia length (**A**) was calculated. Cardiac tissue FITC-conjugated WGA staining (**B**) and quantification of myocyte cross-sectional areas (**C**) were analyzed. The expression of cardiac hypertrophic marker and ANP (**D**) was detected by Western blot. PAS staining (**E**) was performed to examine glycogen accumulation followed by semi-quantitative analysis of staining. Data are presented as the mean ± S.D. n = 6. *P < 0.05 vs. WT; ^#^*P* < 0.05 vs. *db*/*db*.

Hypertrophy plays an important role in diabetes-induced cardiac remodeling. Accordingly, cardiac hypertrophy was induced in *db*/*db* mice compared to WT mice, reflected by the increased ratio of heart weight to tibia length (Fig. 2A), and the enlarged cell size of myocyte cross-sectional areas detected via FITC-conjugated wheat germ agglutinin (WGA) staining (Fig. 2B,C). Furthermore, the expression of atrial natriuretic peptide (ANP), a molecular hypertrophy marker, was also progressively increased in *db*/*db* mice hearts (Fig. 2D). However, all these cardiac hypertrophic changes were significantly prevented by 3 months of treatment with BSE or SFN.

Because accumulation of glycogen is regarded as a characteristic feature of DCM, we examined the cardiac deposition of glycogen using periodic acid-Schiff (PAS) staining. Our results demonstrated a significant increase in glycogen accumulation (indicated by magenta color) in the heart of *db*/*db* mice compared with WT mice. Treatment of BSE and SFN significantly decreased the positive area (Fig. 2E).

**Figure 3:**
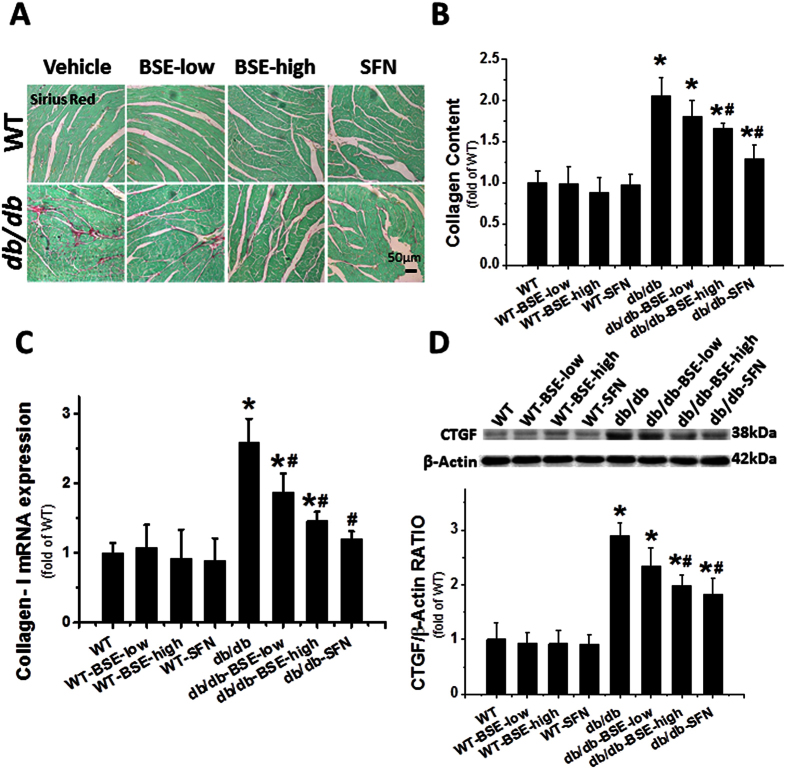
BSE and SFN prevented diabetes-induced cardiac fibrosis. Sirius red staining of collagen (**A**,**B**), real-time PCR of collagen-I (**C**) and Western blot of CTGF (**D**) were detected to estimate the cardiac fibrotic response. Data are presented as the mean ± S.D. n = 6. *P < 0.05 vs. WT; ^#^P < 0.05 vs. *db*/*db*.

As another hallmark feature of cardiac remodeling, fibrosis was evidenced by the accumulation of collagen, detected using the Sirius-red staining (Fig. 3A,B), and confirmed with Collagen-I mRNA expression (Fig. 3C), in the heart of *db*/*db* group compared to control. Cardiac fibrosis was consistent with the change of the pro-fibrotic mediator, connective tissue growth factor (CTGF), measured by Western blot (Fig. 3D). Both BSE and SFN treatment decreased the collagen accumulation and the expression of CTGF. The preventive effect seen in the BSE-high dose group was stronger than the BSE-low dose group, and it more closely aligned to the SFN group with regard to cardiac hypertrophy and fibrosis.

### Like SFN, BSE also up-regulates the expression and function of Nrf2, leading to significant prevention of diabetes-induced oxidative stress and inflammation

**Figure 4:**
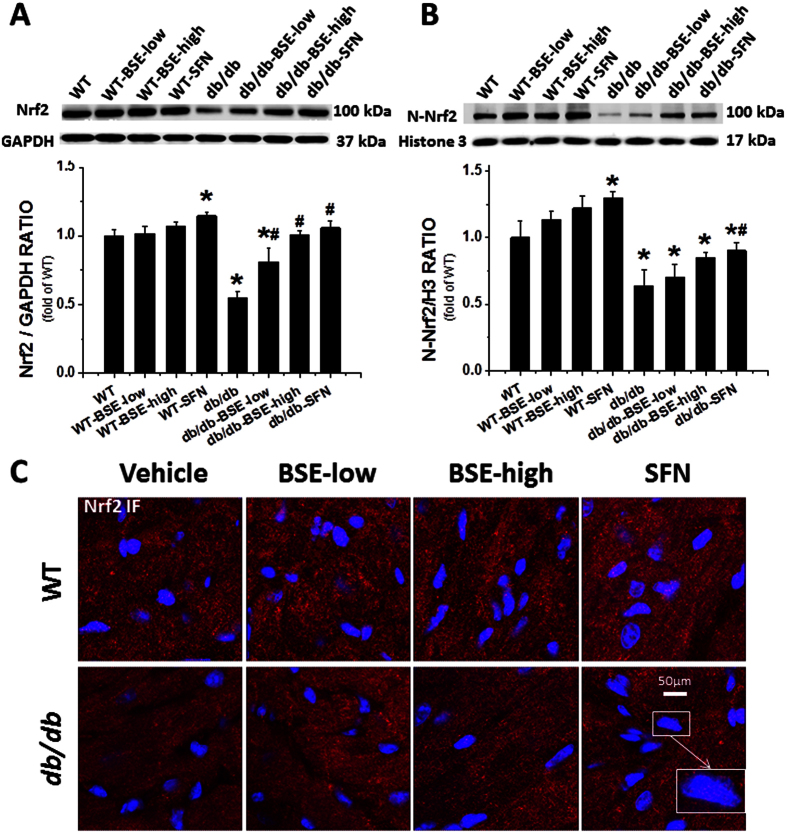
BSE and SFN increased the expression of Nrf2 and induced Nrf2 nuclear translocation in the heart. The expression of Nrf2 was detected by Western blot (**A**). The activation of Nrf2, which was reflected by its nuclear accumulation, was quantified by Western blot (**B**) of the cardiac nucleus protein. IF staining (**C**) was also performed to detect the nucleus accumulation of Nrf2 in hearts with Nrf2 antibody in red and nuclear staining by 4,6-diamidino-2-phenylindole (DAPI) in blue; merged blue and red staining indicates nuclear localization of Nrf2. Data are presented as the mean ± S.D. n = 6. *P < 0.05 vs. WT; ^#^P < 0.05 vs. *db*/*db*.

The above studies confirmed that, like SFN, BSE at the high dose also significantly prevented the development of DCM in *db*/*db* mice compared to WT mice. Mechanistically, SFN’s protection from DCM has been attributed to the activation of Nrf2^29^. Because BSE is a natural source of SFN, we sought to establish whether BSE could protect the heart from diabetes by activating Nrf2. According to the western blot assay (Fig. 4A), Nrf2 expression was slightly increased in the heart of BSE or SFN-treated WT mice. The *db*/*db* group showed a significant down-regulation of Nrf2 levels, which were prevented in BSE- or SFN-treated *db*/*db* mice. Nrf2 plays its transcriptional role in the nucleus. We, therefore, extracted the nucleus and examined the Nrf2 nuclear protein level by Western blot (Fig. 4B) and Immunofluorescent (IF) staining (Fig. 4C). Increased accumulation of nuclear Nrf2 in WT-BSE or SFN group and *db*/*db*-BSE or SFN group was observed, suggesting the potential of Nrf2 activation.

**Figure 5:**
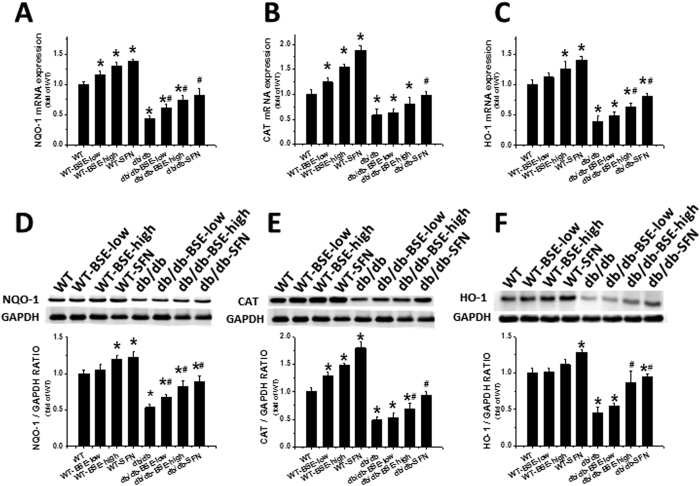
BSE and SFN up-regulated the downstream genes of Nrf2 and associated protein expression levels in the heart. Nrf2 function was determined by the expressions of Nrf2 downstream genes. NQO1, CAT and HO-1 at mRNA (**A**–**C**) and protein levels (**D**–**F**) were observed by real-time PCR and Western blot. Data are presented as the mean ± S.D. n = 6. *P < 0.05 vs. WT; ^#^P < 0.05 vs. *db*/*db*.

We further examined Nrf2 transcription via quantification of its downstream antioxidant gene expression. Both mRNA and protein expression levels of NQO-1 (Fig. 5A,D), CAT (Fig. 5B,E), and HO-1 (Fig. 5C,F), in the hearts of BSE- or SFN-treated WT and *db*/*db* mice were significantly increased. However, these three Nrf2-downstream genes were significantly reduced in the *db*/*db* group, which was in line with the total and nuclear Nrf2 level (Fig. 4B).

**Figure 6:**
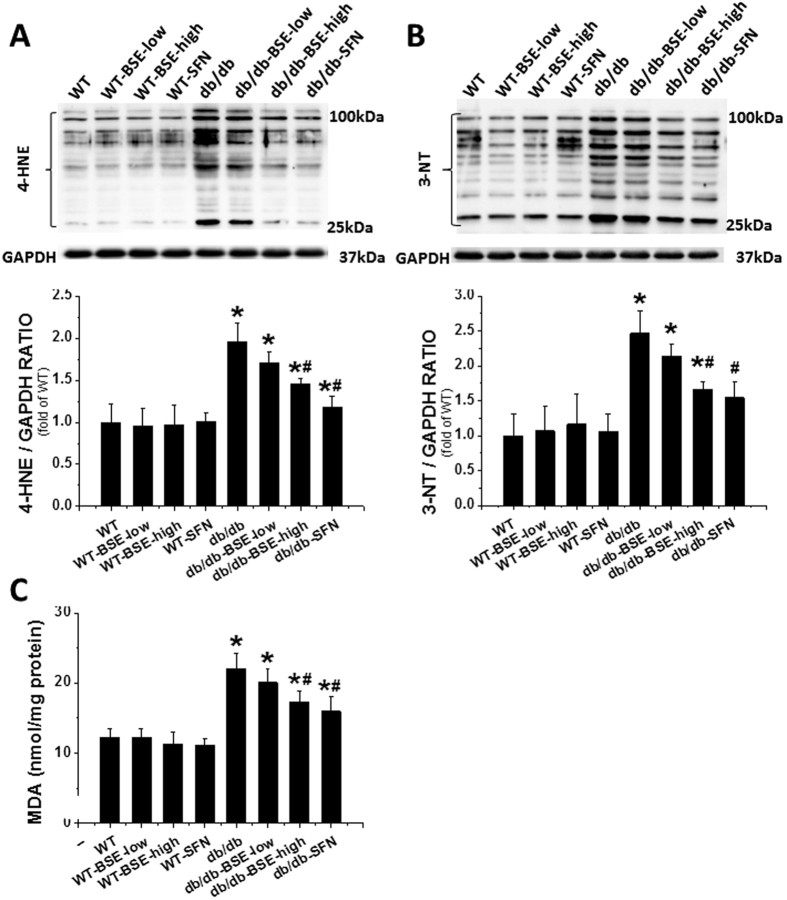
BSE and SFN prevented diabetes-induced cardiac oxidative stress. As oxidative stress markers, the accumulation of 4-HNE (**A**) and 3-NT (**B**) in the heart were evaluated by Western blot. Lipid peroxide accumulation was quantified using MDA (**C**). Data are presented as the mean ± S.D. n = 6. *P < 0.05 vs. WT; ^#^*P* < 0.05 vs. *db*/*db*.

**Figure 7:**
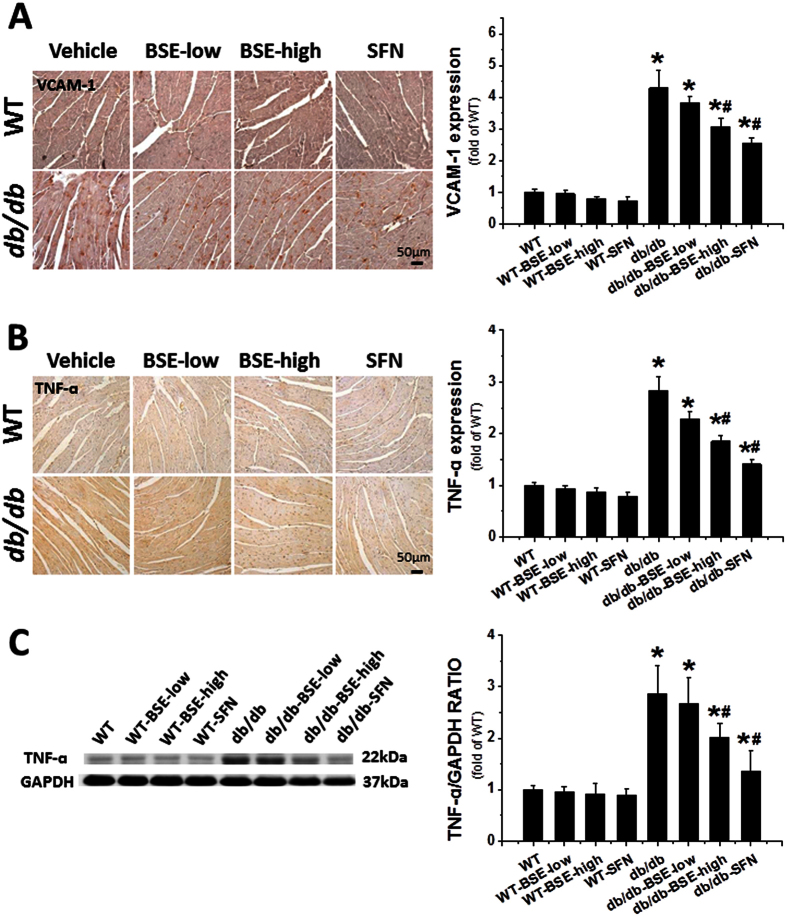
BSE and SFN prevented diabetes-induced cardiac inflammation. The expressions of VCAM-1 (**A**) and TNF-α (**B**) were observed by immunohistochemical staining, with related semi-quantitative analysis of staining. Western blot assay was used to detect the expression of TNF-α (**C**). Data are presented as the mean ± S.D. n = 6. *P < 0.05 vs. WT; ^#^*P* < 0.05 vs. *db*/*db*.

Oxidative stress and inflammation play key roles in diabetes-induced cardiac pathogenesis. Consistent with this finding, the oxidative stress related markers, 4-hydroxy-2-nonenal (4-HNE), 3-nitrotyrosine (3-NT) (Fig. 6A,B) and malondialdehyde (MDA) (Fig. 6C), were significantly increased in the *db*/*db* group; these effects were significantly reduced by BSE or SFN. Similarly, expressions of the inflammatory factors, vascular cell adhesion molecule-1 (VCAM-1) (Fig. 7A) and tumor necrosis factor alpha (TNF-α) (Fig. 7B,C) in hearts, determined by immunohistochemical staining and Western blot assay, were significantly increased in the *db*/*db* group but suppressed in the *db*/*db*–BSE and SFN group.

## Discussion

This study showed, for the first time, that treatment with SFN-rich BSE for 3 months could significantly prevent the pathological process of DCM in the T2DM mice model, and, like SFN, BSE also significantly up-regulated Nrf2 expression and function to prevent diabetes-induced cardiac oxidative stress and inflammation.

In our previous studies, we have shown the protection provided by SFN against DCM via up-regulation of Nrf2 expression and transcription activation, thus, preventing oxidative stress and damage in both the streptozotocin (STZ)-induced type 1 diabetes mellitus (T1DM)^20^ and the T2DM model^29^. Loss of Nrf2 function was found to exacerbate high glucose-induced oxidative stress in the heart, leading to severe cardiac damage^30^. Moreover, we also found the SFN protection from diabetes-induced damage to other organs, including the aorta, testis, and kidney along with the activation of the Nrf2 signaling pathway^31^,^32^,^33^. To develop a clinic-relevant protocol for systemic up-regulation of Nrf2 to prevent the development of DCM, we specifically showed significant prevention using BSE and SFN, along with detail of the up-regulation of cardiac Nrf2 function. Our studies provide the following new information.

In this study, the transgenic *db*/*db* mice were used to establish the development of DCM in the T2DM model. Our previous studies^29^,^31^,^33^,^34^ had revealed the preventive effect of SFN on diabetes-induced organ damage in a mouse model of T2DM that was induced by high fat diet (HFD) feeding plus small dose of STZ. However, in these prior studies, there was no evidence that SFN could prevent the development of DCM in *the db*/*db* mice. Upon review, there is one previous study using *db*/*db* mice to show that SFN treatment could prevent diabetes-induced microvascular dysfunction^35^. The present study not only demonstrated the preventive effect of SFN on the development of DCM in *db*/*db* mice, but it also showed that BSE had a comparable effect to SFN on the prevention of DCM.

Several kinds of broccoli extracts, including BSE, have been utilized in several clinical trials to explore its potential clinical application^10^,^23^,^24^,^25^,^26^,^27^,^28^,^36^. Some of these results have been summarized in recent reviews^37^,^38^. However, the ability of human gut microflora to hydrolyze glucoraphanin and release sulforaphane for absorption in the large intestine varies greatly among individuals^26^,^39^. As a result, the dose of SFN converted from extract was difficult to accurately compare among these separate studies. Furthermore, there was also no information regarding the direct comparison for the SFN absorption into the body between SFN and the SFN-rich broccoli extract administration. In the present study, we have directly compared the effect of BSE at two doses with that of SFN. The amount of BSE given at the low dose was estimated to contribute 0.5 mg/kg SFN, if completely absorbed, which is equivalent to the dose of SFN that was given in our previous study^20^. Although this low dose of BSE showed a significant prevention of the development of DCM, its effect was weaker when compared to that of the SFN at the same dose. Only administration of BSE at the high dose (double dose of BSE-low) could provide a similar effect as that of the SFN on the prevention of DCM. This finding, in fact, is consistent with the conclusion that, in humans, the bioavailability of supplemental free SFN is about four-fold greater than the bioavailability of SFN originating from part of glucoraphanin^40^.

It is worth mentioned that broccoli sprout preparations have been approved to be clinically safe to use in healthy volunteers^25^,^26^,^27^,^28^ for the prevention of chronic obstructive pulmonary disease^10^, asthma^23^,^24^, and even cancers^41^,^42^,^43^. One recent example is a study involving seven patients with schizophrenia that have completed a trial with 3 tablets of SFN, consisting of 30 mg of SFN-glucosinolate per day, for 8 weeks. Results suggested that their cognitive function was significantly improved^44^. In addition, no adverse events were noted which provides additional safety data for SFN-rich broccoli’s product use in this population^44^, and it is in concert with its safety profile when used in health individuals^25^,^26^,^27^,^28^. In terms of the anticancer effects of SFN or broccoli preparations, several early studies have shown its preventive effect on some tumors at varying stages of development, including late stage metastasis. This action is likely via multiple mechanisms, presumably involving the wide range of genes that are regulated by Nrf2, as summarized in a few recent reviews^45^,^46^,^47^. For instance, dietary administration of BSE to rats demonstrated a significant, dose-dependent inhibition of bladder cancer development induced by N-butyl-N-(4-hydroxybutyl) nitrosamine^42^. The incidence, multiplicity, size, and progression of bladder cancer were all inhibited by the extract, while the extract itself caused no histologic changes in the bladder. The inhibition of bladder carcinogenesis by the extract was associated with significant induction of glutathione S-transferase and NQO-1 in the bladder^42^, suggesting its association with Nrf2 activation. Taking these findings together, BSE, along with broccoli’s other products, has great potential for use in the clinical realm for the prevention and treatment of a variety of health conditions.

We are also aware of a few limitations in the present proof-of-concept study. We gave mice BSE-low dose, BSE-high dose and SFN, but we did not evaluate the kinetics and bio-distributions of SFN as done by others^48^. In addition, we did not investigate whether the protective effect of BSE on the heart persisted in Nrf2-knockout mice since its role had been previously defined with SFN. In addition, the extraction method may impact activity and absorption of SFN; therefore, our future study will compare the effects of BSE extracted by different methods in a diabetic mice model.

In summary, we have investigated and showed that, like SFN, BSE can prevent DCM in a transgenic T2DM mouse model via activation of Nrf2 by inhibiting diabetes-induced cardiac oxidative stress and damage as well as inflammation. We also demonstrated that BSE, when used at higher doses, can be used as a natural and safe source of SFN to up-regulate Nrf2 expression and prevent DCM. Similar to its reported beneficial impact when used in patients with other chronic diseases, our results demonstrate that BSE also has promising potential in the treatment of patients with diabetes mellitus.

## Methods

### Animals

Both male type 2 diabetic (FVB.BKS (D)-Leprdb/ChuaJ, *db*/*db*) and FVB WT mice were purchased from the Jackson Laboratory (Bar Harbor, Maine). They were housed in the University of Louisville Research Resources Center at 22 °C with a 12-h light/dark cycle and had free access to standard rodent chow and tap water. All animal experiments were approved by the Institutional Animal Care and Use Committee of the University of Louisville, and performed in accordance with the Guide for the Care and Use of Laboratory Animals published by the US National Institutes of Health (NIH Publication No. 85–23, revised 1996).

BSE (Source Naturals Company, Scotts Valley, CA) or SFN (Sigma-Aldrich, St. Louis, MO) were fed to the mice by gavage every other day for 3 months. Both 5-month old *db*/*db* mice (referred to hereafter as *db*/*db*) and FVB mice (referred to hereafter as WT) were divided into four groups: Vehicle (0.1 ml/10 g), BSE-low dose (equivalent to SFN 0.5 mg/kg), BSE-high dose (equivalent to SFN 1.0 mg/kg), and SFN molecule (0.5 mg/kg) as a positive control. Animals were sacrificed for experimental measurements. Doses of BSE and SFN used were based on an earlier study^20^,^49^.

Since SFN was dissolved in 1% dimethyl sulfoxide (DMSO) and diluted in PBS, BSE was crushed and then made into suspension using Carboxymethyl Cellulose (CMC) and PBS. Mice serving as vehicle control received the same volume of PBS, containing the same concentration of CMC, as that of the BSE groups.

### Intraperitoneal glucose tolerance test (IPGTT)

Glucose tolerance was measured by IPGTT. IPGTT (glucose 2 g/Kg, body-weight) was performed before and after treatment with BSE and SFN, and results shown by calculating the integrated area under the curves.

### Echocardiography

Transthoracic echocardiography (Echo) was performed on mice anesthetized with Avertin using a high-resolution system, equipped with a high-frequency ultrasound probe (RMV-707B), designed for small animals (Vevo 770, Visual Sonics, Canada). Parasternal long-axis and short-axis views were acquired. LV dimensions and wall thicknesses were determined from parasternal short axis M-mode images. The heart rate (HR) of the anesthetized animal was recorded. EF, FS, and LV mass were calculated by Vevo770 software simultaneously (Table 1). Data represent averaged values of 10 cardiac cycles^50^.

### Heart pathology, immunohistochemical and immunofluorescent staining

Following terminal anesthesia with Avertin, hearts were excised and fixed in 10% buffered formalin, and then dehydrated in graded alcohol series, cleared with xylene, embedded in paraffin and sectioned at 5 μm thickness.

For immunohistochemical staining, tissue sections were dewaxed, and then incubated with 1× target retrieval solution (Dako, Carpinteria, CA) for antigen retrieval, followed by 3% hydrogen peroxide and 5% bovine serum albumin for 30 min, respectively. These sections were incubated with primary antibodies overnight at 4 °C. Primary antibodies included TNF-α (Abcam, Cambridge, MA) at 1:100 dilution, VCAM-1 (Santa Cruz Biotechnology, Santa, CA) at 1:100 dilution. Secondary antibodies (1:300–400 dilutions with PBS) were incubated for 1 h in room temperature. Sections were then treated with peroxidase substrate DAB (3, 3-Diaminobenzidine, Vector Laboratories, Burlingame, CA) for coloration and counterstained with hematoxylin.

For immunofluorescent staining, the tissue sections that had been incubated with primary antibody (Nrf2 at 1:100 dilution, Santa Cruz Biotechnology, Santa, CA) were washed with PBS, and then incubated with secondary antibody (Cy3-conjugated donkey anti-rabbit antibody at 1:200 dilution, Jackson ImmunoReserch Laboratories, West Grove, PA), followed by counterstaining with 4, 6-diamidino-2-phenylindole dihydrochloride (DAPI, Sigma-Aldrich). Confocal images were acquired using an Olympus Fluoview FV-1000 confocal scanner coupled to an Olympus 1 × 81 inverted microscope, a PlanApoN 60× objective, and FV-10 ASW2.1 software.

For the myocyte cross-sectional area, sections were stained with fluorescein-conjugated WGA stain (Alexa Fluor-488, Invitrogen). Images were viewed using the confocal microscope.

To detect fibrosis or glycogen accumulation in tissues, sections were stained by Sirius-red staining or PAS staining methods, respectively.

### Real-time qPCR

Hearts were frozen in liquid nitrogen and kept at −80 °C. Total RNA was extracted using TRIzol Reagent (Invitrogen, Carlsbad, CA). RNA concentrations and purities were quantified using a Nano drop ND-1000 spectrophotometer. Reverse transcription was performed using 1 μg of total RNA in 12.5 μL of the solution containing 4 μL 25 mM MgCl_2_, 4 μL AMV reverse transcriptase 5 × buffer, 2 μL dNTP, 0.5 μL RNase inhibitor, 1 μL of AMV reverse transcriptase, and 1 μL of oligo dT primer, which were added with nuclease-free water to make a final volume of 20 μL.

The reaction system was run at 42 °C for 50 min and 95 °C for 5 min. Primers [Hmox1: Mm00516005_m1; NqO1: Mm01253561_m1; CAT: Mm00437229_m1; Collagen- I: Mm01302043_g1; GAPDH: Mm99999915_g1] for PCR were purchased from Thermo Fisher (Grand Island, NY). Real-time qPCR was carried out in a 20 μL reaction buffer that included a 10 μL of TaqMan Universal PCR Master Mix, 1 μL of primer, 3 μL of cDNA and 6 μL nuclease-free water with the ABI 7300 Real-Time qPCR system. Comparative cycle time (CT) was used to determine fold differences between samples^51^.

### Western blot assay

Heart tissues were extracted and separated on 10% SDS-PAGE gels followed by transfer to a nitrocellulose membrane (Bio-Rad, Hercules, CA). The membrane was blocked with a 5% non-fat dried milk for 1 h and then incubated overnight at 4 °C with the following antibodies at different dilutions of 1:1000–1:3000: Nrf2, NQO1, HO-1, CAT, ANP, CTGF, GAPDH and β-actin (Santa Cruz Biotechnology, Santa, CA), 3-NT (Millipore, Billerica, CA), 4-HNE (Alpha Diagnostic International, San Antonio, TX), TNF-α (Abcam, Cambridge, MA). After three washes with Tris-buffered saline (pH 7.2) containing 0.05% Tween 20, membranes were incubated with appropriate secondary antibodies for 1 h at room temperature. Immunoreactive bands were visualized using an enhanced chemiluminescence kit (Bio-Rad). Photos were captured by ChemiDoc Touch Imaging System (Bio-Rad). Protein contents were normalized to that of GAPDH and β-actin.

### Preparation of nuclear protein and western blot analysis of Nrf2

Nuclear protein was extracted using the nuclear extraction kit (Abcam) as follows: small pieces of tissue were weighed and cut. Tissue was homogenized with pre-extraction buffer containing dithiothreitol (DTT) solution, and centrifuged, followed by removal of supernatant. The extraction buffer (containing DTT solution and a protease inhibitor cocktail) was added to the nuclear sediment, and the extract was incubated on ice while being vortexed. Finally, the suspension was centrifuged and supernatant was collected.

### Biochemical measurement of lipid peroxides

To detect the lipid peroxide accumulation in the heart, the tissue protein was tested by measuring thiobarbituric acid reactivity, reflected by the content of MDA formed during acid hydrolysis of the lipid peroxide compound, following the assay procedures described previously^52^. The lipid peroxide level in the heart was expressed in nmol MDA per milligram tissue.

### Statistical analysis

Data were presented as means ± SD (n = 5 at least in each group). Comparisons were performed by one-way ANOVA for the different groups, followed by post-hoc pairwise repetitive comparisons with Turkey test, using Origin 8.0 Lab data analysis and graphing software. Statistical significance was considered as p < 0.05.

## Additional Information

**How to cite this article**: Xu, Z. *et al*. Broccoli sprout extract prevents diabetic cardiomyopathy via Nrf2 activation in *db/db* T2DM mice. *Sci. Rep.* **6**, 30252; doi: 10.1038/srep30252 (2016).

## Figures and Tables

**Table 1:** Protective effect of BSE and SFN on diabetes-induced cardiac dysfunction.

	WT	WT-BSE-low	WT-BSE-high	WT-SFN	*db*/*db*	*db*/*db* -BSE-low	*db*/*db* -BSE-high	*db*/*db* -SFN
IVS; d (mm)	0.63 ± 0.00	0.62 ± 0.02	0.62 ± 0.02	0.63 ± 0.03	0.63 ± 0.03	0.62 ± 0.02	0.61 ± 0.02	0.63 ± 0.04
LVID; d (mm)	3.74 ± 0.02	3.74 ± 0.06	3.76 ± 0.01	3.79 ± 0.02	4.09 ± 0.08*	4.12 ± 0.07*	4.05 ± 0.06*	4.06 ± 0.05*
LVPW; d (mm)	0.85 ± 0.05	0.82 ± 0.05	0.80 ± 0.04	0.85 ± 0.06	0.84 ± 0.03	0.82 ± 0.04	0.78 ± 0.03	0.79 ± 0.04
IVS; s (mm)	1.01 ± 0.03	0.99 ± 0.02	0.96 ± 0.07	1.00 ± 0.02	1.00 ± 0.02	1.00 ± 0.02	0.98 ± 0.02	0.99 ± 0.03
LVID; s (mm)	2.06 ± 0.05	2.04 ± 0.03	2.07 ± 0.06	2.09 ± 0.03	2.83 ± 0.08*	2.65 ± 0.07*,#	2.50 ± 0.05*,#	2.48 ± 0.04*,#
LVPW; s (mm)	1.29 ± 0.02	1.30 ± 0.01	1.28 ± 0.02	1.28 ± 0.03	1.30 ± 0.02	1.28 ± 0.02	1.27 ± 0.02	1.30 ± 0.04
LV Vol; d (mm)	59.52 ± 0.60	59.54 ± 2.18	60.22 ± 0.61	61.62 ± 0.61	73.93 ± 2.59*	74.89 ± 2.45*	71.98 ± 2.40*	72.56 ± 2.17*
LV Vol; s (mm)	13.72 ± 0.71	13.42 ± 0.53	13.88 ± 0.98	14.19 ± 0.54	31.43 ± 0.74*	25.72 ± 1.81*,#	22.37 ± 1.00*,#	21.97 ± 0.89*,#
%EF	76.94 ± 1.16	77.44 ± 1.35	76.94 ± 1.70	76.98 ± 0.65	57.93 ± 1.32*	65.67 ± 2.21*,#	68.88 ± 1.94*,#	69.72 ± 0.78*,#
%FS	44.88 ± 1.10	45.36 ± 1.33	44.90 ± 1.60	44.95 ± 0.61	30.18 ± 0.92*	35.72 ± 1.66*,#	38.16 ± 1.59*,#	38.84 ± 0.64*,#
LV Mass (mg)	94.31 ± 4.69	88.55 ± 1.29	90.37 ± 4.10	92.42 ± 6.81	109.27 ± 4.86*	106.74 ± 5.08*	101.33 ± 0.96*,#	101.10 ± 3.95*,#

Notes: IVS: interventricular septum; LVID; d: left ventricular internal diastolic diameter; LVID; s: left ventricular internal systolic diameter; LVPW: left ventricular posterior wall; EF: ejection fraction; FS: fractional shortening; LV vol; s: left ventricular end systolic volume; LV vol; d: left ventricular end diastolic volume; LV mass: left ventricular mass. Data are presented as means ± SD. *p < 0.05 vs. WT group; ^#^p < 0.05 vs. *db*/*db* group.
